# Findings From a Service Evaluation on Use of Intramuscular Clozapine in a Large NHS Mental Health Trust From 2017 to 2023

**DOI:** 10.1192/bjo.2026.11585

**Published:** 2026-06-30

**Authors:** Indu Surendran, Maryam Al-Talabani, Ed Silva, Inti Qurashi, Rachel Walsh

**Affiliations:** 1 Mersey and West Lancashire NHS Teaching Hospital, Prescot, United Kingdom; 2 University Hospitals Sussex NHS Foundation Trust, Worthing, United Kingdom; 3 Merseycare NHS Foundation Trust, Prescot, United Kingdom

## Abstract

**Aims:**

Administration of clozapine is challenging in treatment-resistant schizophrenia (TRS) patients who refuse to take it orally; enforced treatment with clozapine, either naso-gastrically (NG) or intramuscularly (IM), may be required in such situations. There are a few existing case-series and reports in this area. This service evaluation aimed to add to this pool of data and to understand the illness profile of patients who received IM clozapine, tolerability, their longer-term outcomes etc.

**Methods:**

The evaluation was of a retrospective longitudinal observational nature and included all patients under the care of Mersey Care NHS Foundation Trust who were prescribed IM clozapine from 01/01/2017 till 31/12/2023. Pseudonymised data was derived from the patient records.

**Results:**

The sample included a total of 30 patients. All patients suffered from chronic psychotic illnesses. Average duration of psychotic illness prior to being prescribed IM clozapine was 19 years. 80% of patients were in seclusion or segregation at the time of IM clozapine being prescribed. Over 40% patients suffered from cardiometabolic or other physical comorbidities. 21 of the patients (70%) belonged to the initiation group and were prescribed IM clozapine to initiate and establish the patient eventually on oral clozapine. 10 (47.6%) of them accepted oral clozapine without receiving any injections. 9 of the patients (30%) belonged to the maintenance group and were prescribed IM clozapine as an adjuvant to maintain their compliance. A total of 428 doses of IM clozapine were given in the study to 17 patients, and each dose was given as 1-2 injections. 7 patients (41.2%) who received IM clozapine developed injection-site nodules or lumps without abscess; average number of doses received was 43. 10 patients (52%) required restraints by PMVA-trained staff; these were uneventful. Around 90% of patients in the initiation group were established on oral clozapine at end of follow-up. Around 2/3rds of patients in the initiation group who established on oral clozapine had seclusion/ segregation terminated, moved to lower securitymeasures and were on clozapine at time of discharge.

**Conclusion:**

Administration of IM clozapine is fairly safe and well tolerated and it should be prescribed in patients earlier in their course of treatment-resistant-schizophrenia where oral compliance is not possible: nearly half of initiation group accepted oral clozapine without a single IM injection, improvement in patient outcomes was noted and majority were established on oral clozapine. Rotation of injection sites is recommended to prevent and avoid injection site nodules.

